# A Piezoelectric–Piezoresistive Coupling Electric Field Sensor for Large Dynamic Range AC Electric Field Measurements

**DOI:** 10.3390/s24030902

**Published:** 2024-01-30

**Authors:** Xiaobo Wang, Bofeng Luo, Rongbo Zhang, Yang Song, Yaqian Zhao, Chengtao Luo

**Affiliations:** 1 School of Electronic Information and Electrical Engineering, Shanghai Jiao Tong University, Shanghai 200240, China; 022035910015@sjtu.edu.cn (X.W.); zhangrongbo@sjtu.edu.cn (R.Z.); chantchant@sjtu.edu.cn (Y.S.); 2 Grid Digital Grid Research Institute, China Southern Power, Guangzhou 510000, China; luobf@csg.cn (B.L.); zhaoyq3@csg.cn (Y.Z.)

**Keywords:** electric field sensor, piezoelectric–piezoresistive coupling, power systems, high linearity, large dynamic electric field range

## Abstract

We propose a piezoelectric–piezoresistive coupling electric field sensor capable of performing large dynamic range AC electric field measurements. The electric field sensor utilizes direct coupling between piezoelectric (PE) materials and piezoresistive (PR) strain gauges, in conjunction with an external signal conditioning circuit, to measure AC electric fields effectively. We verified the feasibility of the scheme using a finite element simulation, fabricated a prototype of the electric field sensor, and characterized the properties of the prototype. The testing results indicate that the sensor exhibits an ac resolution of 50 V/m and a linear measurable electric field range of 0 to over 200 kV/m, which keeps the linearity at less than 0.94% from 1 Hz to over 5 kHz. Furthermore, the sensor also has advantages, such as a small size and low power consumption. The sensor can enhance the comprehensive observability and measurability of digital power grids.

## 1. Introduction

**Figure 1:**
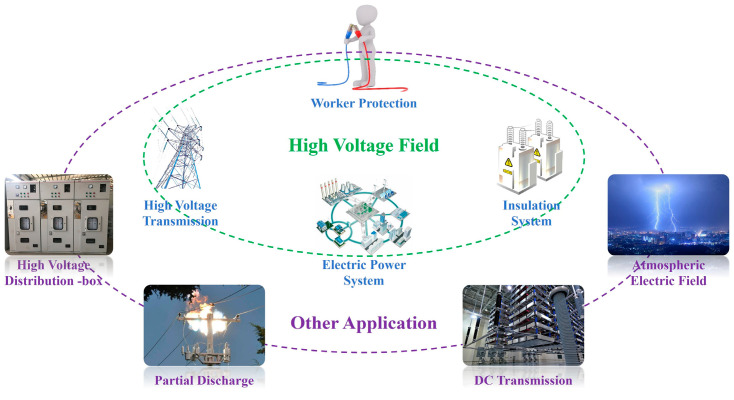
Application fields of E-sensors.

With the development of electric power systems and Internet of Things (IoT) technology, detection networks can be formed by arranging or internally integrating electric field sensors (E-sensors) to achieve real-time monitoring of power systems [1]. This aids in the identification of potential faults, ensuring the safe and efficient operation of a power system and its equipment. This requires E-sensors to meet the characteristics of miniaturization, low power consumption, high electric field resolution, and high linearity [2]. As shown in Figure 1, in addition to their widespread applications in high-voltage fields, E-sensors also have extensive applications in areas such as atmospheric and DC transmission, partial discharge, and high-voltage distribution.

At present, miniature E-sensors can be categorized into four types based on their measurement principles: electro-optic effect-based miniature E-sensors, induction charge-based miniature E-sensors, static force-based miniature E-sensors, and inverse piezoelectric effect-based miniature E-sensors [3].

**Figure 2:**
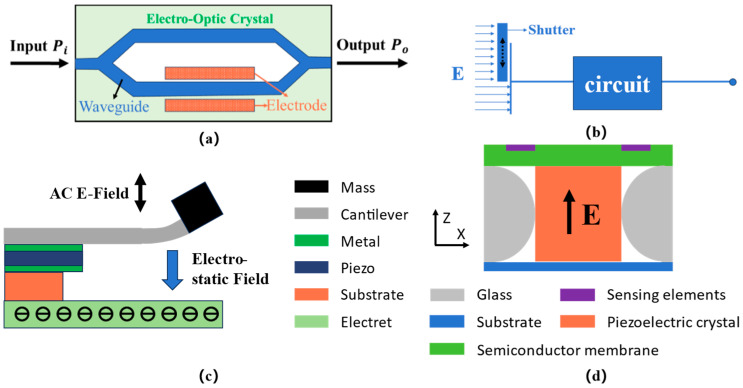
Schematic diagrams of E-sensors: (**a**) principle of the electro-optic effect-based E-sensor from Bulmer et al. [4]; (**b**) principle of the induction charge effect-based E-sensor from P. S. Riehl et al. [22]; (**c**) principle of the static force effect-based E-sensor from X. Wang et al. [15]; (**d**) principle of the inverse piezoelectric effect-based E-sensor from F. Xue et al. [20,23].

The electro-optic effect refers to the phenomenon where certain isotropic transparent materials exhibit optical anisotropy under the influence of an electric field, leading to changes in their refractive indices. Sensors based on this effect have an early start and mature technology, offering the advantages of high resolution and wide measurement bandwidth. As shown in the Figure 2a. Electro-optic effect-based E-sensors can achieve a resolution of 1 mV/m–1 V/m and a measurement bandwidth of 300 MHz–10 GHz and above [4,5]. However, due to the use of complex optical components, these sensors have a high environmental sensitivity, a reliance on light sources, and larger sizes, which may result in slower response times in extreme environments [6,7,8].

The most typical example of induction charge-based E-sensors is the field mill electric field meter, which integrates shielding plates, sensing plates, photoelectric encoders, and motors [9,10]. Due to the integrated rotating shielding plates, these sensors can measure DC electric fields, but traditional field mill electric field meters are large and have low measurement accuracy. MEMS technology-based field mill E-sensors have achieved miniaturization, with dimensions of about 5 mm × 5 mm and an electric field resolution of up to 600 V/m [1]. As shown in the Figure 2b. Through optimization of the driving method, the electrode direction, the vibration direction, and the electrode shape of MEMS induction charge E-sensors, the current range of these sensors can reach 0–5 kV/m, with a resolution of 42 V/m [11,12,13]. Compared to electro-optic effect-based E-sensors, MEMS induction charge E-sensors are smaller and less expensive, but due to their limited sensing area they have lower resolution, and maintaining long-term operational stability is a major challenge.

As shown in the Figure 2c. Static force coupling-based miniature E-sensors measure electric fields through the deformation and displacement of sensitive elements under static forces [14,15]. These sensors can achieve a resolution of 20 V/m and an electric field detection range of 312 kV/m–700 kV/m, with a high signal-to-noise ratio [16,17,18]. However, their cut-off frequency is often limited by the structural resonance frequency. Inverse piezoelectric effect-based miniature E-sensors have been a new direction in E-sensor research in recent years.

In recent years, with the development of piezoelectric materials, E-sensors based on the inverse piezoelectric effect have been developed rapidly. Based on the inverse piezoelectric effect of PE materials, these sensors characterize electric fields through mechanical deformation. As shown in the Figure 2d. Combined with MEMS technology, they can achieve miniaturization and low power consumption. The resolution of these sensors can reach tens of V/m, with a testing range at the mV/m level [19,20]. These sensors are suitable for large-scale deployment, offering good frequency characteristics and high-temperature stability. However, enhancing the resolution of these sensors and improving micro-nano fabrication technologies are the main challenges at this stage. In 2022, the J. Wu team from Xi’an Jiao Tong University proposed a high-resolution electric field sensor based on a piezoelectric bimorph composite [21]. This electric field sensor has a large detection range and a high resolution for electric fields. Due to its operation in the bending mode of piezoelectric materials, it is capable of testing low-frequency electric fields. Nevertheless, the sensor’s test bandwidth and linearity are also subject to certain limitations.

In this paper, we propose an E-sensor based on the inverse piezoelectric effect and PE–PR coupling. We validated the feasibility of this approach through theoretical verification and optimized the design of the E-sensor structure using finite element simulation software. Then, we completed the prototype fabrication using PZT-5H and a P-type silicon. Using the experimental system, the property evaluation was performed, and the results demonstrated that the measurable electric field ranges from 0 to over 300 kV/m, the measurable frequency ranges from 1 Hz to over 5 kHz, and linearity can reach less than 1%. The E-sensor we propose can meet the power grid system’s requirements for a compact sensor size, a large dynamic range, and high linearity.

## 2. Theoretical and Simulation Analysis

The sensing principle of the E-sensor proposed in this paper was realized through the coupling of a PE material (PZT-5H) with semiconductor strain gauges (P-type silicon). The PE crystal and PR strain gauge constitute the sensitive elements of the E-sensor. Due to the inverse piezoelectric effect of the PE material, mechanical strain is generated within the material when it is subjected to an electric field.

In this design, as the PE material operates, the equation is as follows [24]:

(1) $$\begin{array}{c} \begin{array}{c} S_{h}=s_{hk}^{E}T_{k}+{\tilde{d}}_{jh}E_{j}\\ D_{i}=d_{ik}T_{k}+\varepsilon_{ij}^{T}E_{j} \end{array} \end{array}$$

where ***S*** is the strain tensor; $s^{E}$ is the compliance tensor under a constant electric field; T is the stress tensor; d is the piezoelectric strain constant matrix; D is the electric displacement vector; $\varepsilon^{T}$ is the permittivity tensor under constant stress; E is the electric field vector; *h*, *k* = 1, 2, …, 6; and *i*, *j* = 1, 2, 3. In the direct coupling of the PE material with the PR strain gauge, assuming no coupling loss, the strain experienced by the PR strain gauge is equal to that of the PE material. As a semiconductor PR material, the resistance change in the P-type silicon strain gauge is a result of strain-induced alterations in the material’s band structure, thereby changing its resistivity [25]. The relationship between the electric field and the current J inside the PR strain gauge is as follows:

(2) $$\begin{array}{c} E=\rho \cdot J+∆\rho \cdot J \end{array}$$

ρ is the resistivity, and ∆ρ is the induced change in resistance. Both ρ and ∆ρ are second-order matrices. The change in resistance is related to the stress $T_{k}$:

(3) $$\begin{array}{c} ∆\rho =\left(\pi_{l}+\pi_{t}\right)\cdot T_{k}\cdot l_{pr}\cdot w_{pr} \end{array}$$

$\pi_{l}$ represents the longitudinal PR coefficient; $\pi_{t}$ represents the transverse piezoresistive coefficient (SI: ${\mathrm{Pa}}^{-1}\;\Omega m$), which is a material property; $l_{pr}$ is the length of the PR strain gauge; $w_{pr}$ is the width of the PR strain gauge; and $t_{pr}$ is the thickness of the PR strain gauge. We used the following formula for calculating resistance:

(4) $$\begin{array}{c} R=\rho \frac{l_{pr}}{S} \end{array}$$

where *S* is the cross-sectional area of the PR strain gauge. By combining the above equations, we obtain the following:

(5) $$\begin{array}{c} ∆R=\left(\pi_{l}+\pi_{t}\right)\frac{\left(D_{i}-\varepsilon^{T}E_{j}\right)}{d_{ik}}\frac{l_{pr}^{2}}{w_{pr}^{2}t_{pr}} \end{array}$$

Formula (5) shows that the change in resistance of the PR strain gauge depends on its size and material parameters. When the parameters of the PR strain gauge are fixed, the PE material has a crucial impact on the change in resistance of the PR material. In this case, with the piezoelectric constant being negative, a piezoelectric material with better piezoelectric properties is required.

**Figure 3:**
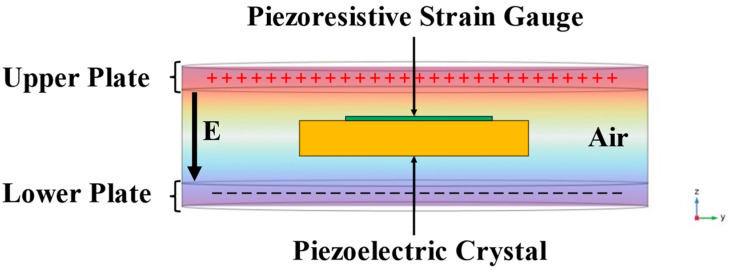
Finite element model of the E-sensor’s sensitive element.

The PE vibrator is directly coupled with the PR strain gauge using epoxy resin. In the simulation software, multi-physics modules, such as solid mechanics, electrostatics, current, and circuitry, were utilized to achieve the simulation of the sensitive element. Figure 3 presents the model established in the finite element software, corresponding to the actual setup. The model simulates a uniform parallel electric field generated between two copper plates by applying voltage excitation from top to bottom. The sensitive element is simulated at the center of the electric field to conduct the electric field excitation.

Based on the vibrational mode dimensional parameters provided by IEEE [26], we used finite element simulation software to compare the output characteristics of the PE–PR coupled structure under different vibrational modes, specifically the signal longitudinally poled length extensional ($k_{33}\;\mathrm{mode}$), the signal transversely poled length extensional ($k_{31}\;\mathrm{mode}$), and the flexural vibration mode.

**Figure 4:**
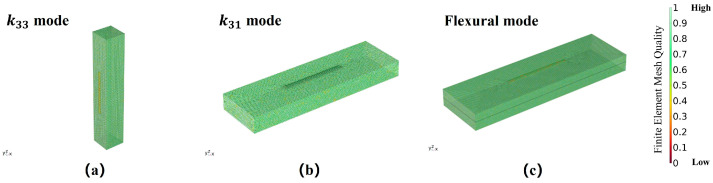
Finite element model mesh division quality. (**a**) $k_{33}$ mode; (**b**) $k_{31}$ mode; (**c**) flexural vibration mode. The legend represents the quality of the finite element mesh, which is applicable to (**a**–**c**).

In the finite element software, the division of the mesh is crucial for the results of simulations. The completed mesh is characterized by skewness, which refers to the degree of deviation from a standard element. Compared to the standard equiangular ideal element, some elements may have a high or low degree of tilt, which can be characterized by skewness. The best characterization quality for a mesh is one, indicating the optimal element in the selected mesh quality assessment. Figure 4 represents the quality of mesh division under three different vibration modes: $k_{33}$ mode, $k_{31}$ mode, and flexural vibration mode. According to the results of the drawings, the overall mesh element quality is close to one, indicating that the mesh quality meets the requirements of the simulation.

**Figure 5:**
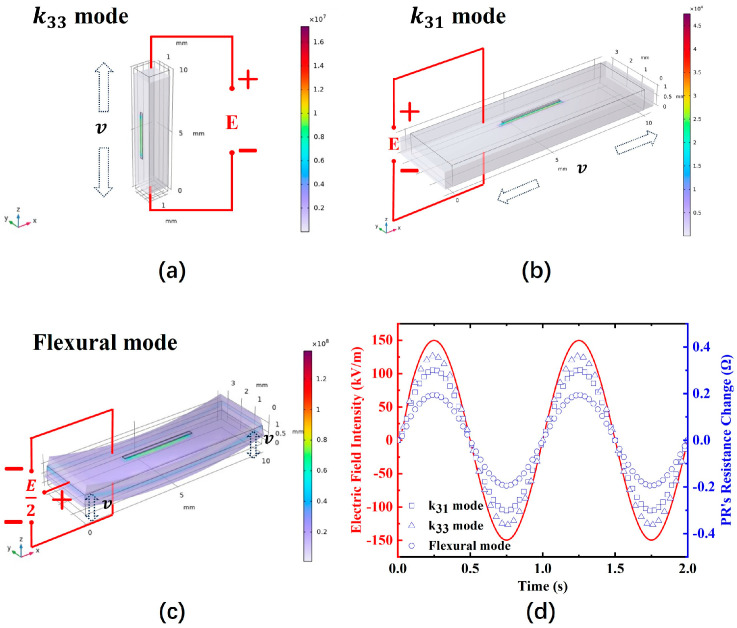
Various vibration modes of the sensitive element and comparisons of the E-sensor’s sensitive element: (**a**) the output characteristics of the sensitive element in $k_{33}$ mode; (**b**) the output characteristics of the sensitive element in $k_{31}$ mode; (**c**) the output characteristics of the sensitive element in flexural mode; (**d**) a comparison between the input signal and the output results of the sensitive element.

Figure 5a shows the simulation results under the $k_{33}$ mode, with $l:w:t=\sqrt{3}\;\mathrm{mm}:\;\sqrt{3}\;\mathrm{mm}:10\;\mathrm{mm}$. Figure 5b presents the simulation results under the $k_{31}$ mode, with l:w:t=10 mm: 3 mm:1 mm. Figure 5c illustrates the simulation results under the flexural vibration mode, with l:w:t=10 mm: 3 mm:1 mm. As shown in Figure 5d, under the same PE volume and identical electrical field excitation boundary conditions, the sensitive element operating in $k_{33}$ mode exhibits superior output characteristics. This is because the direction of the applied electric field is the same as the polarization direction of the PE crystal. It is typically the most sensitive direction in piezoelectric materials. Consequently, the inverse piezoelectric effect is most pronounced in this orientation. However, for PZT-5H materials, piezoelectric elements operating in $k_{33}$ mode encounter challenges in polarization. Therefore, taking all factors into consideration, we chose to use $k_{31}$ as the operational mode for the sensor element.

**Figure 6:**
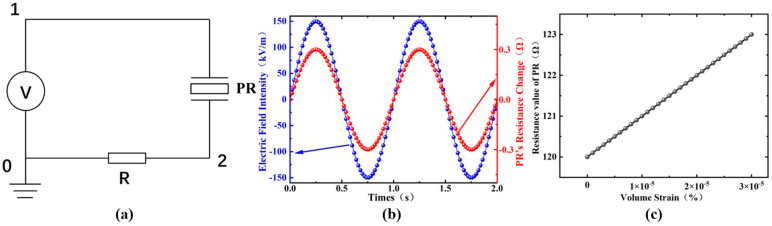
(**a**) Finite element simulation circuit model; (**b**) simulation results of the resistance change in the PR strain gauge; (**c**) relationship between the volumetric strain and resistance change in the PR strain gauge.

In the simulation software, the circuit module was used to connect the PR strain gauge with a fixed resistor, enabling the measurement of the loop current, as shown in Figure 6a. This was achieved by connecting different nodes to integrate the PR strain gauge with the fixed resistor. Under the excitation of an alternating electric field, a simulation of the resistance change in the PR strain gauge was realized, as depicted in Figure 6b, which showed a resistance change of approximately 0.3 Ω. In conjunction with a Wheatstone bridge and an external signal conditioning circuit, the conversion of electric field strength to voltage signal was achieved. As illustrated in Figure 6c, we also simulated and calculated the relationship between the strain of the PR strain gauge and the change in resistance. After fitting, a linear relationship was observed. However, in actual coupling, a nonlinear relationship exists due to material characteristics, coupling coefficients, hysteresis, and other issues, which are not discussed in this paper.

In this section, we validated the theoretical foundation of the PE–PR coupled E-sensor and analyzed the feasibility of this approach through finite element simulation. In the following section, we will discuss the fabrication of the sensor prototype and the construction of the testing system.

## 3. Sensor Fabrication and Experimental System Setting

We completed the fabrication of the E-sensor prototype, the design of the signal conditioning circuit, and the construction of the experimental system. In the next section, we will conduct tests on typical indicators of the E-sensor.

We selected a 120 Ω single-crystal silicon PR strain gauge and a PE crystal of PZT-5H. The PE and PR were coupled together with epoxy resin and then heated in an oven at 60 °C for 6 h to ensure a firm coupling. The sensitive element was placed inside the packaging shell, and the leads of the PR were drawn out with wires, resulting in the fabrication of the E-sensor prototype.

**Figure 7:**
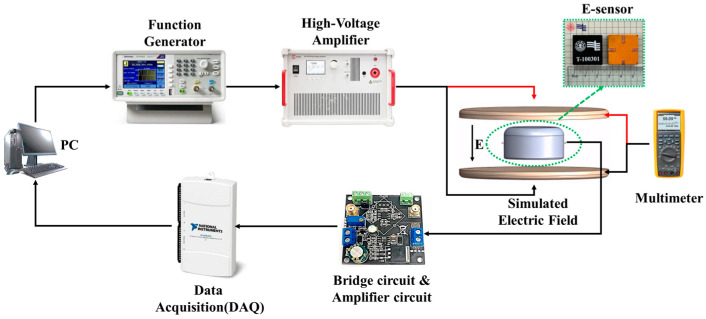
E-sensor testing system and E-sensor prototype.

We designed an electric field experimental system for testing the E-sensor prototype, as shown in Figure 7. The voltage signal generated (AFG1022, Tektronix, Beaverton, OR, USA) by the signal generator is amplified to the copper plates using a high-voltage amplifier (ATA-7050, Aigtek, Xi’an, China), creating a uniform electric field between the plates. The sensor is connected to an external signal conditioning circuit, which filters and amplifies the signal before sending it to the acquisition card (Ni USB-6210, National Instruments, Austin, TX, USA). The signal is then collected and processed on a PC. At the same time, during the electric field test, in order to monitor the size of the electric field in real-time, a multimeter is connected to the upper and lower copper plates to measure the voltage between the two copper plates. When the distance between the copper plates is fixed, the ratio of the voltage across the copper plates to the distance between them represents the intensity of the generated electric field.

For the signal conditioning circuit part of the E-sensor, we utilized an AD623 instrumentation programmable amplifier to achieve differential amplification. At the same time, the Wheatstone bridge component was also integrated into the circuit module, with a built-in 5 V power supply module directly powering the bridge module. The total power consumption of the entire system circuit was only 60 mW.

## 4. Sensor Testing and Results Discussion

We conducted measurements of the detection range, linearity, hysteresis, and other parameters for the E-sensor prototype.

This metric included two ranges: the electric field intensity range and the electric field frequency range. Detecting low-frequency electric field signals is a current challenge. Starting from 1 Hz, the electric field frequency was gradually increased, and tests were conducted for electric field strengths of 0–200 kV/m. After stabilizing each point for 30 s, three data points were recorded.

**Figure 8:**
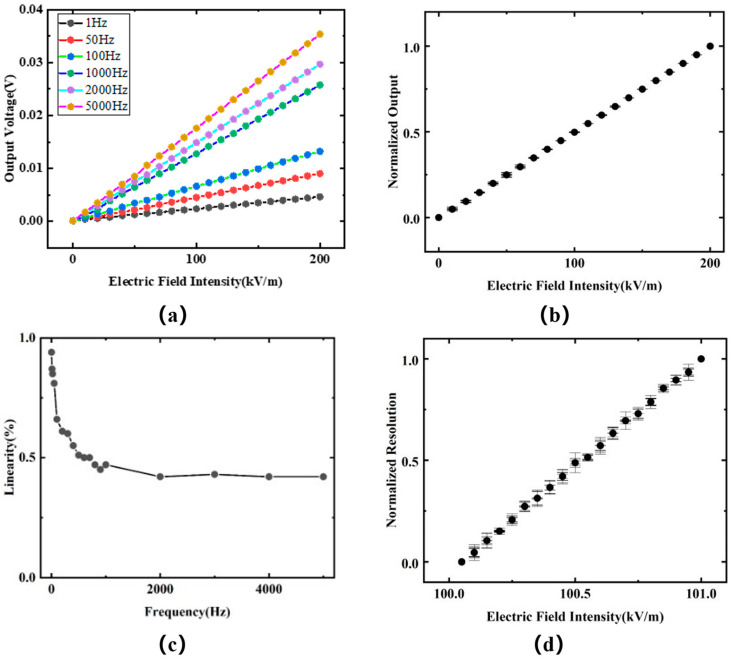
Test results of the E-sensor prototype. (**a**) The electric field response of the sensor prototype at typical frequencies; (**b**) normalized results of the output characteristics of the E-sensor; (**c**) linearity curves of the E-sensor under typical electric field strengths and at different frequency excitations; (**d**) normalized results of the resolution of the E-sensor prototype. I. Detection range.

**Figure 9:**
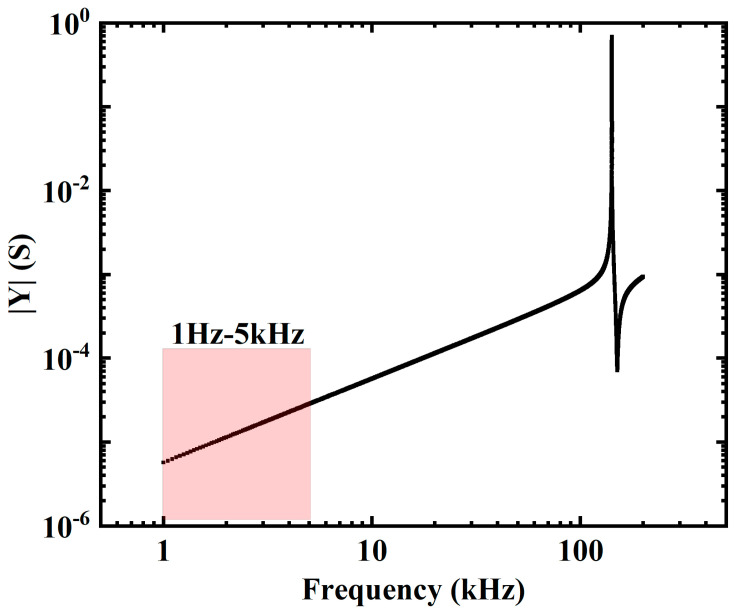
Admittance curve of the E-sensor’s sensitive element.

As shown in Figure 8a, within the specified range, the output of the E-sensor is linearly related to the intensity of the stimulating electric field. Additionally, the output of the sensor positively correlates with the frequency of the electric field. As shown in Figure 9, we calculated the admittance curve of the sensitive element using finite element simulation software. Within the applied frequency range, the admittance value gradually increased with the increase in frequency, which was determined by the material characteristics of the PE crystal. This inevitably introduced non-linearity in the sensor’s output. However, we ensured that its resonant frequency was more than five times higher than the upper limit of the application frequency, thereby minimizing the impact caused by its material characteristics as much as possible.

As shown in Figure 8b, we normalized the output results of the E-sensor prototype under different frequency fields, eliminating the impact of frequency on the prototype. We then averaged all normalized results, considering the electric field output at typical frequencies as the error. The results shown in Figure 8b characterize the E-sensor as having good output stability and linearity.

It is important to note that 1 Hz–5 kHz is merely the linear operating range of the high-voltage amplifier. Beyond 5 kHz, the amplification factor of the high-voltage amplifier is subject to certain limitations. When the frequency is below 1 Hz, the testing system generates unavoidable interference, leading to significant systematic errors in the measurements. Based on the comprehensive performance of the E-sensor prototype, it is estimated that 1 Hz–5 kHz is not the maximum detection frequency range of this sensor.

### 4.1. Linearity

This value reflects the degree of output linearization of the E-sensor within the test range, also known as non-linear error. First, uniformly select m calibration points within the specified measurement range (including the upper and lower limits of the test range). Starting from the lower limit of the measurement range $E_{0}$, steadily increase the electric field to each calibration point. After stabilization, read the sensor’s output value, continuing until the upper limit of the measurement range is reached:

$$\xi_{L}=\frac{\left|∆Y_{L\cdot max}\right|}{Y_{FS}}\times 100\%$$

where $∆Y_{L\cdot max}$ is the maximum deviation of the actual output characteristic curve of the sensor from the reference line, $Y_{FS}$ is the full-scale output of the sensor, and $\xi_{L}$ is the linearity of the sensor. According to the above formula, the linearity results at various frequencies were less than 0.94%.

### 4.2. Hysteresis

The hysteresis error of the sensor is the fundamental cause of backlash. The calculation formula is

$$\xi_{H}=\frac{{\left|\bar{Y_{U𝑖}}-\bar{Y_{D𝑖}}\right|}_{max}}{Y_{FS}}\times 100\%$$

where $\bar{Y_{U𝑖}}$ is the average value of the forward stroke test value at the ith calibration point, $\bar{Y_{D𝑖}}$ is the average value of the reverse stroke test value at the ith calibration point, and $\xi_{H}$ is the hysteresis error of the sensor. According to the measurement results and the above formula, the hysteresis error of the self-developed sensor was less than 2%.

### 4.3. Resolution

Based on the specified frequency range, we adjusted the electric field input. An electric field of $E_{0}+∆E_{step}$ (where $E_{0}$ is any electric field within the measurement range and $∆E_{step}$ is the specified resolution) was applied as the baseline, and the sensor’s output value $y_{c0}$ was recorded. The electric field was then increased to $E_{0}+2\times ∆E_{step}$, and the sensor’s output value $y_{c2}$ was recorded. After reapplying the electric field of $E_{0}+∆E_{step}$ and reducing the electric field to $E_{0}$, the sensor’s output value $y_{c1}$ was recorded. It should satisfy the following condition: if $y_{c1}<y_{c0}<y_{c2}$, then the resolution is $∆E_{step}$. Through calculations, our E-sensor prototype was found to have a resolution of 50 V/m. We characterized the sensor within an electric field intensity range of 100 kV/m to 101 kV/m, and the test data were characterized through normalization, as shown in Figure 8d. The results indicate that, based on the normalization and error estimation of the E-sensor, there were some fluctuations in the error. However, it was able to meet the condition $y_{c1}<y_{c0}<y_{c2}$.

Based on the test parameters of the E-sensor prototype, a comparison was made with the specifications of existing E-sensors. The comparison results are shown in Table 1:

Due to different technical routes, various types of E-sensors have different advantages. Electric field sensors based on the electro-optic effect are capable of measuring high-frequency and large dynamic electric fields. MEMS electric field sensors based on the inverse piezoelectric effect have the advantage of a fast response and extremely small size. Meanwhile, capacitive electric field sensors, made using the bending mode of piezoelectric materials, can measure both DC and AC, and offer a large electric field strength detection range and a high resolution. The advantage of our proposed electric field sensor lies in its ability to detect low-frequency and large dynamic electric fields, while also providing excellent linearity and resolution. Additionally, its fabrication process is simple and the cost is low.

Summarizing the test results, the design of this PE–PR coupled E-sensor has been validated. The E-sensor prototype can measure electric field frequencies ranging from 1 Hz to 5 kHz and intensities ranging from 0 to 200 kV/m. Within the test range, it achieves a resolution of 50 V/m, a hysteresis error < 2%, and maintains a linearity of ≤0.94%. In this paper, the primary focus is on validating the principle of the PE–PR electric field sensor. There are several directions for further optimization of the sensor. For instance, the sensor could be fabricated using superior PE materials, such as PMN-PT piezoelectric single crystals with higher piezoelectric coefficients. Additionally, multiple PR strain gauges could be arranged on the PE crystal to form various types of bridge circuits, thereby further enhancing the overall output of the sensor.

## 5. Conclusions

In this paper, we propose a PE–PR coupling E-sensor for AC electric field measurement. The sensor utilizes PE–PR coupling between a $k_{31}$ mode PZT-5H vibrator and a P-type silicon, which was validated by theoretical and finite element analyses. The E-sensor was then fabricated, and the experimental results show that it has good linearity, a high resolution, and can detect a wide dynamic range of AC electric fields (resolution: <50 V/m, electric field: 0–200 kV/m, 1 Hz–5 kHz; linearity: ≤0.94%; and hysteresis error: <2%).

Geared towards power grid systems, its excellent performance, as well as its small size and low power consumption, make it ideal for integration into large-scale network applications and smart grid infrastructure.

## Figures and Tables

**Table 1:** Comparison of the performance between different types of E-sensors.

Properties	Electro-Optic Effect E-Sensor [4,5,27]	MEMS Inverse Piezoelectric Sensor [19,20,28]	Capacitive-Sensing Sensor [21]	PE–PR Coupling E-Sensor in This Work
Resolution	1 mV/m–1 V/m	20 V/m	10 V/m	50 V/m
Linearity	<1%	<2%	/	<0.94%
E-filed intensity range	0–1500 kV/m	0–100 kV/m	0–300 kV/m	0–200 kV/m
Frequency range	300 MHz–10 GHz	DC-AC (MHz)	DC-AC	1–5 kHz
Power consumption	Low	Low	Low	60 mW
Size	Medium	Very Small	Small	Small
Characteristics	High frequency, large electric field range.	Correspondingly fast, high stability, small size.	High sensitivity, DC measurement, large electric field range.	Low frequency measurement, large electric field range, high linearity, simple preparation.

## Data Availability

Data is contained within the article.
